# Supplementary material for: Modulation of Stemness and Differentiation Regulators by Valproic Acid in Medulloblastoma Neurospheres
Source: Cells. 2025 Jan 7;14(2):72. doi: 10.3390/cells14020072 (PMC11763699; doi:10.3390/cells14020072)
Supplement: Supplementary file 1 [file cells-14-00072-s001.zip › cells-3337326-supplementary.pdf]

Supplementary Information

Modulation of Stemness and Differentiation Regulators by Valproic Acid in Medulloblastoma Neurospheres

Natália Hogetop Freire, Alice Laschuk Herlinger, Júlia Vanini, Matheus Dalmolin, Marcelo A. C. Fernandes, Carolina Nör, Vijay Ramaswamy, Caroline Brunetto de Farias, André Tesainer Brunetto, Algemir L. Brunetto, Lauro José Gregianin, Mariane da Cunha Jaeger, Michael D. Taylor and Rafael Roesler

Supplementary Table S1. Forward and reverse primers used for RT-qPCR amplification.

Gene	Primer Forward (5′-3′)	Primer Reverse (5′-3′)
ACTB	AAACTGGAACGGTGAAGGTG	AGAGAAGTGGGGTGGCTTTT
CDKN1A	ACTCTCAGGGTCGAAAACGG	CTTCCTGTGGGCGGATTAGG
ENO2	AGCCTCTACGGGCATCTATGA	TTCTCAGTCCCATCCAACTCC
NES	GATCGCTCAGGTCCTGGAAG	GGGGTCCTAGGGAATTGCAG
MYC	TACAACACCCGAGCAAGGAC	AGCTAACGTTGAGGGGCATC
PTRG	TGCATGCAAGATTCATCCCACCC	TGCAATACTCCTGTTGGTAGGGC A
SOX2	CAGCTCGCAGACCTACATGA	GGGAGGAAGAGGTAACCACAG
TUBB3	CTCAGGGGCCTTTGGACATC	CAGGCAGTCGCAGTTTTCAC
TP53	ACCTATGGAAACTACTTCCTGAA A	CTGGGAGCTTCATCTGGACC

Supplementary Table S2. Primers targeting the promoter region of *TP53* in ChIP analysis.

Gene	Primer Forward (5′-3′)	Primer Reverse (5′-3′)
TP53	TTTAGCGCCAGTCTT GAGCA	GTATCTACGGCACCA GGTCG

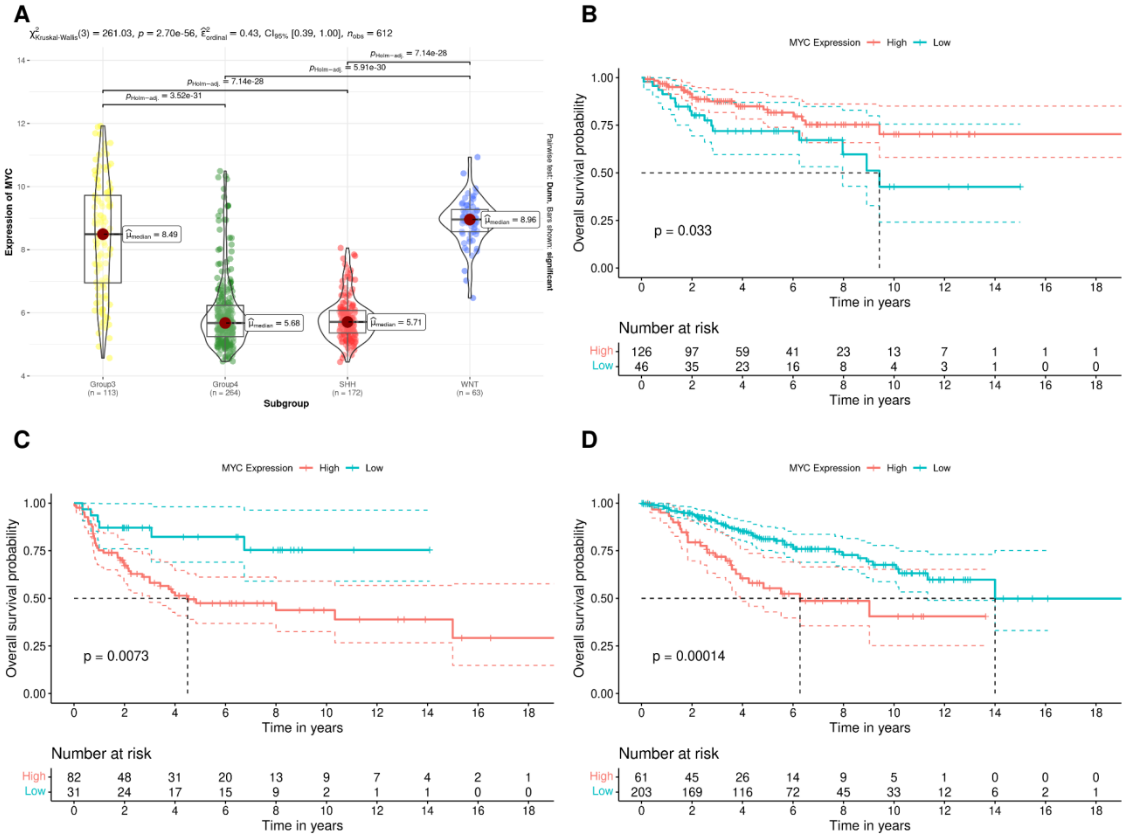

Supplementary Figure S1. *MYC* gene expression and association with patient prognosis across different molecular subgroups of human MB. **(A)** Transcript levels in group 3 (n = 113), group 4 (n = 264), SHH (n = 172), and WNT (n = 64). **(B-D)** OS of patients bearing MB tumors with high or low levels of *MYC* expression classified into different molecular subgroups. Analyses were performed as described in Materials and Methods; *P* values are indicated in the figure.

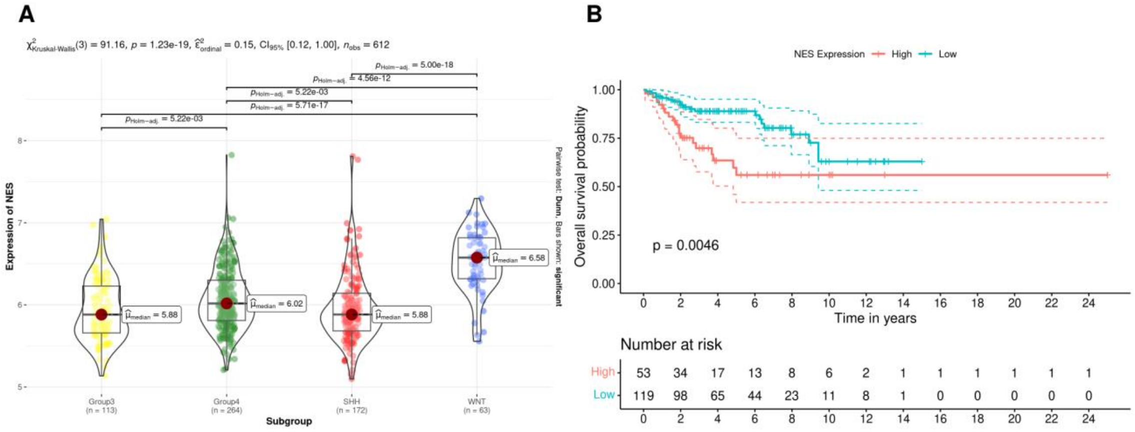

Supplementary Figure S2. *NES* gene expression across different molecular subgroups of human MB and association with prognosis in patients with SHH MB. (**A)** Transcript levels in group 3 (n = 113), group 4 (n = 264), SHH (n = 172), and WNT (n = 64). (**B)** OS of patients bearing SHH MB tumors with high or low levels of *NES* expression. Analyses were performed as described in Materials and Methods; *P* values are indicated in the figure.

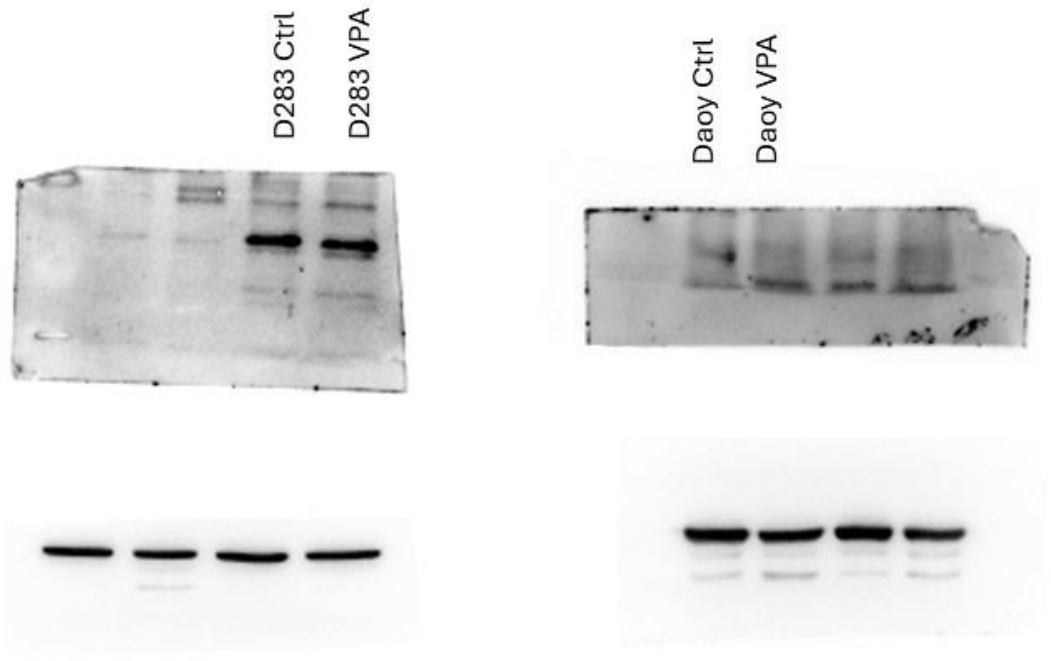

Supplementary Figure S3. Uncropped blots for the experiment shown in Figure 2C describing the Western blot analysis of p21 protein in D283 and Daoy MB cells.
